# Rest in Heart Disease

**Published:** 1902-08-30

**Authors:** 


### REST IN HEART DISEASE.

In the course of a clinical lecture at the Liverpool Royal Infirmary Dr. Richard Caton[1] discussed the causes which prevent the restoration of normal valve function in rheumatic endocarditis. As others have done before, he contrasted the perfect recovery which is met with after rheumatism of the joints with the degree of crippling which so often follows the same disease when it affects the endocardium, and pointed to the cause of this in the fact that in the one case a degree of rest could be obtained which was in the other impossible. As he justly said, suppose for a moment that we were able to cause an acutely rheumatic knee to be flexed and extended 80 or 100 times per minute during the whole period of the attack, is there any probability that resolution would follow? The negative answer which must be given to this question is perhaps the key to the pessimistic view so commonly taken of the future of a case in which endocarditis has developed in the course of rheumatism. Dr. Caton shows, however, to how large an extent permanent evil consequences may be avoided if every care be taken throughout the whole course of the attack to give the heart the greatest amount of rest that is practicable. Pain and fever are to be subdued as rapidly as possible, for which purpose salicylates are useful, absolute quiet is to be enjoined, the patient must lie with the head low, no excitement is to be permitted, he is in every way to be made as comfortable as possible, sleep is to be encouraged, and a light unstimulating diet is to be given. Dr. Caton also advocates gentle blistering of those areas of skin which have a special nerve connection with the heart, thus imitating the effect of a blister over a rheumatic joint, and the administration of moderate doses of sodium iodide.

[1] Lancet, August 23.

